# Smartphone-based study reminders can be a double-edged sword

**DOI:** 10.1038/s41539-024-00253-7

**Published:** 2024-06-21

**Authors:** Lea Nobbe, Jasmin Breitwieser, Daniel Biedermann, Garvin Brod

**Affiliations:** 1 Education and Human Development, DIPF | Leibniz Institute for Research and Information in Education, Frankfurt am Main, Germany; 2 IDeA-Center for Research on Individual Development and Adaptive Education of Children at Risk, Frankfurt am Main, Germany; 3 Information Center for Education, DIPF | Leibniz Institute for Research and Information in Education, Frankfurt am Main, Germany; 4 Department of Psychology, Goethe University, Frankfurt am Main, Germany

**Keywords:** Human behaviour, Education

## Abstract

Reminders are a popular feature in smartphone apps designed to promote desirable behaviors that are best performed regularly. But can they also promote students’ regular studying? In the present study with 85 lower secondary school students aged 10–12, we combined a smartphone-based between- and within-person experimental manipulation with logfile data of a vocabulary learning app. Students were scheduled to receive reminders on 16 days during the 36-day intervention period. Findings suggest that reminders can be a double-edged sword. The within-person experimental manipulation allowed a comparison of study probability on days with and without reminders. Students were more likely to study on days they received a reminder compared to days when they did not receive a reminder. However, when compared to a control group that never received reminders, the effect was not due to students studying more frequently on days with reminders. Instead, they studied less frequently on days without reminders than students in the control group. This effect increased over the study period, with students becoming increasingly less likely to study on days without reminders. Taken together, these results suggest a detrimental side effect of reminders: students become overly reliant on them.

## Introduction

Effective, self-regulated learning means taking responsibility for one’s own learning^1^. It is a key competence of the 21^st^ century because it enables individuals to keep up with social, technological, and economic change^2^. However, students often struggle with the application of effective learning techniques^3–7^. The self-regulation skills facilitating their self-directed learning need to be taught and cultivated^8^. Distributing study time over multiple sessions (i.e., distributed practice), for example, is a well-established learning strategy that is effective for many different types of material and is particularly fruitful for long-term retention^9–11^, but students –and especially younger ones–often fail to apply it^3^. Instead, they often improvise their study method and evaluate strategies based on how much they learned short-term^12,13^. Our goal, therefore, is to investigate effective methods to help lower secondary school students integrate distributed practice into their self-study. Lower secondary school is a sensitive period for the development of self-regulated learning because students at this level are expected to take more responsibility for their own learning, for example when studying vocabulary^14^.

Digital technology can be used to support self-regulated learning. Mobile technologies allow for widely available interventions that can support successful self-study^15,16^. To date, many digital interventions for self-regulated learning have been designed for computer-based learning environments^17^. Within such learning environments, interventions tend to be context-specific, while mobile interventions also lend themselves to teaching transferable skills applicable to a wide range of materials, contents, and tasks^15^. However, existing mobile interventions are often designed to be used at the beginning and during study sessions^18^. This may be too late for distributed practice. While students could decide not to study all the material at once during the study session, it requires the will to study in a distributed manner and the foresight to remember to study early and often enough to have time for distributed practice. This means that promoting distributed practice may require support outside of study sessions.

A commonly used idea in technology designed to change behavior are nudges^19^. Nudges refer to “any aspect of the choice architecture that alters people’s behavior in a predictable way without forbidding any options or significantly changing their economic incentives”^20^. Importantly, according to Thaler and Sunstein’s definition, nudges do not have to encourage behavior that aligns with the default behavior, preference, or even benefit of the individual being nudged^21^. This is in stark contrast to the promotion of self-regulated learning activities. It is paramount that interventions used to promote beneficial study behaviors align with students’ intentions and are implemented together with them.

One simple way to support distributed practice in everyday life is through reminders that can be delivered to people’s smartphones (for instance via push notifications). These reminders can remind recipients of information they already know or convey additional new information. They therefore actively support decision-making. They refocus attention on both the task and the benefits of task completion^21^ and are a cost-effective and unobtrusive form of intervention that can be provided repeatedly in order to increase the effectiveness of one-off instruction^16^. Providing reminders that are continuously presented on devices that students carry in everyday life allows for the integration of support for distributed practice into everyday life.

The literature on study reminders for students is quite scarce. However, positive effects of reminders on studying have been found^22,23^ and they have been used with teachers and students’ parents^24^. Reminders are more extensively used in the field of mobile health (mHealth) research. Within mHealth, reminders are used to promote various desirable behaviors best performed regularly such as sufficient water intake, adherence to chronic medication, and physical activity^25–28^. For reminders that focus on less frequent or even one-off events such as college enrollment or parking tickets, interindividual differences in the effectiveness of reminders have been found, with low-responders being more likely to come from disadvantaged groups^21,29^. In recent years, efforts have also focused on how the positive effects of reminders can be enhanced, through the timing and frequency with which they are sent^30^ but also through the amount of information included^31^.

However, reminders may also have negative side effects. For instance, they can evoke feelings of guilt for having to be reminded^32^. When sent too often or at inappropriate times, they can be an annoyance and possibly lead to disengagement^32^. Even when users like the reminders they get, they can still have detrimental consequences. Stawarz and colleagues^33^ found that, while reminders supported repetition, they hindered habit formation. This suggests an overreliance effect, where the beneficial behavior is only shown when reminders are present but is not transferred to situations without reminders. Such an effect could run counter to the intended effect of our intervention in promoting distributed practice and is therefore taken into account in the present study.

To investigate the effects of reminders on study frequency, we conducted a smartphone-based between-^34^ and within-person experimental manipulation^35^. This means that reminders were alternated based on the day of the study to compare students’ study probability on days with and without reminders. While all participating young students watched an explanatory video on distributed practice and its benefits, only some received additional reminders of the video’s contents on about 16 of the 36 days of the intervention (pseudorandomly assigned). Study behavior was measured objectively and with high ecological validity using logfiles of a vocabulary learning app that the students used. Thus, we were able to avoid the biased perceptions and memories that can sometimes influence self-reports^1^. The schedule of reminder presentation in the Reminder Group, in combination with a Control Group of students who never received reminders, allowed us to unravel both positive effects and possible “hidden costs” of reminders. Based on previously demonstrated positive effects of reminders^16,28^, our preregistered hypotheses were as follows:

H1: Students in the Reminder Group have a higher likelihood of studying with the vocabulary learning app on days with reminders than on days without reminders.

H2: Students in the Reminder Group study more frequently with the vocabulary learning app than students in the Control Group.

H3: Students in the Reminder Group perform better in the vocabulary tests than students in the Control Group.

H4: The more frequently students study with the vocabulary learning app, the better they perform in the vocabulary tests.

## Results

### Descriptive statistics

**Fig. 1:**
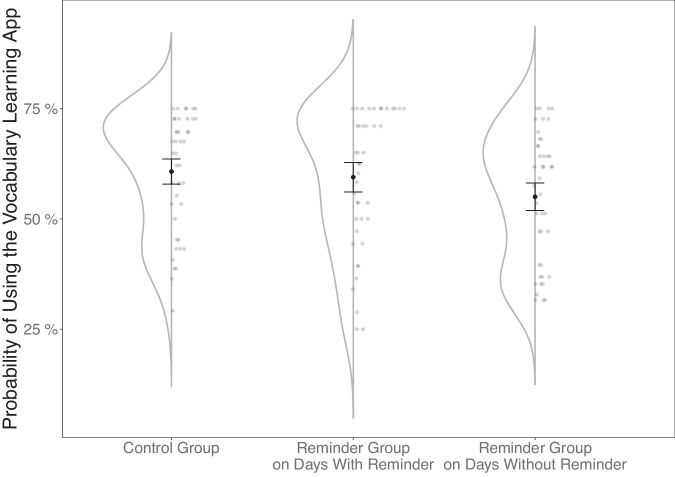
Descriptive data for the experimental groups and day types. The (sub)group means and distribution of data. Bars represent the 95% confidence interval. The Reminder Group data is split up into data from days with and without reminders.

As shown in Fig. 1, students in the Control Group studied, on average, on 71% (*SD* = 27%) of the study days (i.e., about 26 out of 36 days). Overall, students in the Reminder Group studied on 62% (*SD* = 29%) of the days (i.e., about 22 days). When they received a reminder, they studied with a probability of 69% (*SD* = 31%). Their study probability for days they did not receive a reminder was 60% (*SD* = 29%).

Students in the Control Group used the study app on 77% (*SD* = 24%) and students in the Reminder Group used the study app on 76% (*SD* = 23%) of all days during the intervention phase. This difference was not significant (*t*(82) = 0.04, *p* = 0.972, 95% *CI* [−0.10, 0.10]). Students in the Reminder Group on average missed 4.14 reminders (*SD* = 4.23, range: 0–14).

For students who took tests, our inclusion criteria for vocabulary tests led us to include one to four tests per participant (*M* = 2.86, *SD* = 1.21). On average, students reached test scores of 76% (*SD* = 17%) with an average test length of 39.55 vocabulary words (*SD* = 14.92). Students in the Control Group took 3.08 tests on average (*SD* = 1.13) and those in the Reminder Group took 2.65 (*SD* = 1.09). Their overall test results were 76% for the Control Group (*SD* = 18%) and 77% for the Reminder Group (*SD* = 16%).

Additionally, we looked into students’ responses to questions on distributed practice and their study behaviors they answered on the introductory day of the study (see Table 1). We focused on questions on distributed practice and students’ study behavior with regards to the intent and ability to start to study. Table 2 shows the means, standard deviations, and correlations for those items and the extent of students’ use of the vocabulary learning app cabuu and our study app. Usage of the two apps was highly correlated. None of the questions looked into predicted the usage of the study app. However, the belief that only studying before the test would be sufficient to know the vocabulary on the test (negative belief) was negatively correlated to the usage of the vocabulary learning app.

**Table 1:** Chosen items taken from the introductory session

Abbreviation	Item Text	Response format
positive belief	The more often I study vocabulary with cabuu, the better I can remember it. [Je öfter ich in cabuu Vokabeln lerne, desto besser werde ich sie mir merken können.]	1 = does not apply at all, 2 = rather not apply, 3 = rather applies, 4 = fully applies
negative belief	Even if I study my vocabulary shortly before the exam, I will know it well on the test. [Auch wenn ich die Vokabeln erst kurz vor dem Test lerne, werde ich sie im Test gut können.]	1 = does not apply at all, 2 = rather not apply, 3 = rather applies, 4 = fully applies
value	It is important to me to study with cabuu every day if possible. [Es ist mir wichtig, in cabuu möglichst jeden Tag Vokabeln zu lernen.]	1 = does not apply at all, 2 = rather not apply, 3 = rather applies, 4 = fully applies
expectancy	I think I will manage to study vocabulary (almost) every day in cabuu. [Ich glaube, ich werde es schaffen, (fast) jeden Tag in cabuu Vokabeln zu lernen.]	1 = does not apply at all, 2 = rather not apply, 3 = rather applies, 4 = fully applies
study frequency	Imagine you’re taking a vocabulary test in a week’s time and you haven’t studied yet. How many days will you study in the week leading up to the test? [Stell dir vor, du schreibst in einer Woche einen Vokabeltest und hast noch nicht gelernt. Wie viele Tage lernst du dann in der Woche bis zum Test?	1 = does not apply at all, 2 = rather not apply, 3 = rather applies, 4 = fully applies
self-control	I find it easy to make myself study, even if I would rather be doing something else. [Es fällt mir leicht, mich zum Lernen zu bringen, auch wenn ich lieber etwas anderes tun würde.]	1 = does not apply at all, 2 = rather not apply, 3 = rather applies, 4 = fully applies

**Table 2:** Means, standard deviations, and correlations with confidence intervals for questionnaire data and app usage

Variable	*M*	*SD*	2	3	4	5	6	7	8
2.cabuu used	24.34	9.96							
3. study app used	27.94	8.31	0.78^a^						
			[0.68, 0.85]						
4. positive belief	3.71	0.46	0.06	0.06					
			[−0.16, 0.27]	[−0.15, 0.27]					
5. negative belief	2.27	0.78	−0.22^a^	−0.16	−0.01				
			[−0.41, −0.01]	[−0.36, 0.06]	[−0.22, 0.21]				
6. value	3.54	0.61	0.05	0.08	0.58^a^	0.01			
			[−0.17, 0.26]	[−0.14, 0.29]	[0.42, 0.70]	[−0.20, 0.23]			
7. expectation	3.58	0.52	0.05	0.05	0.37^a^	0.11	0.51^a^		
			[−0.16, 0.26]	[−0.17, 0.26]	[0.17, 0.54]	[−0.11, 0.32]	[0.33, 0.65]		
8. study frequency	3.46	1.23	0.12	0.06	0.22^a^	0.02	0.27^a^	0.14	
			[−0.10, 0.32]	[−0.15, 0.27]	[0.01, 0.41]	[−0.20, 0.23]	[0.06, 0.46]	[−0.08, 0.34]	
9. self-control	2.56	0.87	0.03	0.14	−0.09	0.00	0.20	0.17	0.34^a^
			[−0.18, 0.24]	[−0.07, 0.34]	[−0.29, 0.13]	[−0.21, 0.21]	[−0.01, 0.40]	[−0.05, 0.37]	[0.13, 0.51]

*Note*. ^a^ indicates significance. The exact items and their translation can be found in Table 1. “Cabuu used” reflects both studying and test taking.

### Students Are More Likely to Study Vocabulary After Receiving a Digital Study Reminder

In line with our first hypothesis, we found a significant positive effect of Reminder on students’ likelihood to study vocabulary on a given day (*b* = 0.57, 95% *CI* [0.28, 0.826], χ^2^(1) = 14.39, *p* < 0.001). On average, students in the Reminder Group were more likely to study on days they received a reminder (*OR* = 1.77, 95% *CI* [1.32, 2.37]). We also explored changes in the effect of the reminders over time and found that the effect of the reminders increased with time (*b* = 0.31, 95% *CI* [0.01, 0.62], χ^2^(1) = 3.78, *p* = 0.046). Overall, these results suggest that reminders had a short-term positive effect on students’ frequency of vocabulary learning and that reminders become increasingly important for starting to study over time.

### Students Receiving Digital Study Reminders Show Signs of Overreliance

In contrast to our second hypothesis, the Reminder and Control Group did not differ in their likelihood to study on a given day (*b* = −0.64, 95% *CI* [−1.41, 0.14], χ^2^(1) = 2.56, *p* = 0.106). In line with this finding, but in contrast to our initial hypotheses, students in the Reminder Group also did not outperform students in the Control Group in the vocabulary tests (*b* = 0.05, 95% *CI* [−0.38, 0.47], χ^2^(1) = 0.05, *p* = 0.829).

We followed up on these surprising findings by splitting up the Reminder Group into days when they received reminders and days without reminders. For days with reminders, we found no significant differences in study frequency compared to the Control Group (*b* = −0.02, 95% *CI* [−0.92, 0.89], χ^2^(1) = 0.00, *p* = 0.960). However, when looking at the days no reminders were presented, we found that students in the Reminder Group were significantly less likely to study vocabulary than students in the Control Group (*b* = −0.79, 95% *CI* [−1.53, −0.05], χ^2^(1) = 4.23, *p* = 0.037, *OR* = 0.45, 95% *CI* [0.22, 0.95]). One possible mechanism causing such a pattern could be an overreliance on the reminders, i.e., students only studying when they actually receive a reminder.

**Fig. 2:**
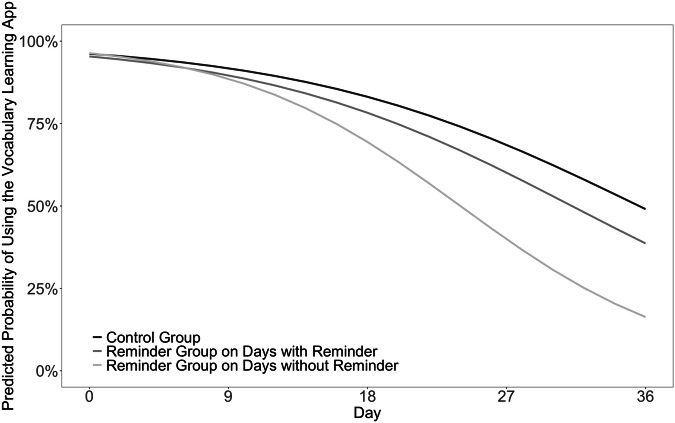
Changes in probability to study over time. The change in study probability over the course of the study for the Control Group and Reminder Group on days with and without reminder.

If students in the Reminder Group become overly reliant on the reminders, the detrimental effect of reminders should increase over time. What we found is a non-significant trend (see Fig. 2): for days when they did not receive reminders, their likelihood to study decreased more rapidly than for students in the Control Group (i.e., day type x day interaction; *b* = −0.05, 95% *CI* [−0.09, 0.00], χ^2^(2) = 6.86, *p* = 0.061). No such trend was found for days with reminders (*b* = −0.01, 95% *CI* [−0.06, 0.05], χ^2^(2) = 6.86, *p* = 0.815). Taken together, these results point to a potential detrimental side effect of reminders: students become overly dependent on them.

## Discussion

Reminders, often in the form of push-notifications, are a popular feature in smartphone apps designed to promote desirable behaviors that are best performed regularly. They were shown to increase water intake, adherence to chronic medication, and step count^25–28^. However, reminders also come with possible negative effects such as annoyance or hinderance of habit formation^32,33^, and their effects on students’ study behavior are unclear.

The present intervention study, which combined a smartphone-based between- and within-person experimental manipulation trial with logfile data from a vocabulary learning app, suggests that reminders can be a double-edged sword: they do work in that students were more likely to study on days on which they received a reminder compared to days without reminders. However, in contrast to our hypotheses, the comparison with a control group without reminders showed that this result was not due to an increased learning frequency of the students with reminders. Instead, the effect was a result of these students learning less frequently when they did not receive reminders. Moreover, for days without reminders, these students studied less and less over time—a pattern that was much milder among the students in the Control Group. Taken together, these results suggest potential detrimental side effects of regular reminders: students might become overly dependent on them.

The promise of reminders lies in the ease with which they can be sent. Whether in addition to one-off interventions or on their own, reminders can be sent repeatedly in people’s daily lives. In this way, they can promote the recall and repetition of desirable behaviors^33^. However, simple repetition of a desired behavior in reaction to a reminder does not mean the desired behavior is performed automatically. Its performance might instead be dependent on the presentation of reminders, i.e., a reliance on the reminders is created. A reliance on reminders could be present, for instance, when students choose to use reminders as a tool, setting them themselves or tying their studying to a reminder schedule they know. If the schedule works, students should then be reminded to study however often necessary. In turn, blind trust in reminders sent out without their schedule being known to students speaks for overreliance.

However, any reliance on digital reminders is at odds with the goal of promoting students’ self-regulated learning. As the term self-regulated learning implies: The goal should be to enable students to choose and apply appropriate learning strategies such as distributed practice making them “masters of their own learning”^36^. Ideally, then, reminders should fade out over time, while the frequency of studying should remain the same. The findings of the current intervention study suggest that simple reminders to study do not help to achieve this goal.

For students to learn in a distributed manner without reminders, studying must become a habit. Habits are “automatic behavioural responses to environmental cues”^37^. This automaticity results from the repetition of an action in a consistent cue context which in turn leads to a cue-response association in memory^38^. With increased automaticity, the behaviors turned habits require less and less self-control and conscious decision-making^38,39^. Once formed, habits are not sensitive to fluctuating motivation or rewards^38^. Our results suggest that repeated reminders to study do not facilitate habit formation. On the contrary, the fact that students in the Reminder Group studied less on days without prompts than students in the Control Group suggests that they might even interfere with the process of tying behavioral responses to environmental cues.

While repeated smartphone reminders to study encourage repeated studying, they do not necessarily tie it to a consistent context. Therefore, a first step in forming a habit might be to identify an appropriate recurring situation to cue studying. This cue then needs to be linked to the goal-directed action that should be performed in contingency with it. This can be achieved with plans or commitments^38^. A specific form of plan are implementation intentions that link a goal-directed action to a specified cue^40^. Such implementations intentions allow for learners to express their own preferences regarding the timing of an action and therefore can create plans that fit into the context of their daily lives^41^. They have been successfully used to foster the habit of dental flossing^42^. For vocabulary learning, Breitwieser and colleagues^43^ found that prompting students to form an implementation intention mitigated the decrease in learning frequency over time. Additional reminders of the implementation intentions and the benefits of distributed practice further increased the frequency of studying. Combining the results of Orbell and Verplanken^42^ and Breitwieser and colleagues^43^ with ours, it seems that how reminders are designed is crucial. Gravert^44^ posed the questions of whether the way reminders are written can improve their effectiveness. Beyond the way they are phrased, they might be more effective when helping to form a habit through establishing easily identifiable study cues in students’ daily lives rather than in their phones.

For an educational intervention to be ultimately useful, it must translate into improved educational outcomes. We did not find differences in vocabulary test performance between groups, however. While this is in contrast to our hypotheses, it would have been surprising to find differences in test performance given that the groups did not differ in their frequency of studying. Moreover, the vocabulary test data was very noisy because several students did not adhere to the intervention study procedure, testing themselves at unexpected times and more or less often than they were asked to. This made students’ study-test cycles very hard to compare.

Some of our results should be generalized only with caution. First, the time trend analyses were exploratory and should be confirmed in future studies. Second, our sample consisted of German lower secondary school students aged 10–12. Younger students often have more difficulties with self-regulation^7^. In addition, younger students also deal with simpler material than older students. Different material or its amount may also make different learning strategies appropriate^3^. It is therefore unclear whether our findings can be generalized to older students and other learning strategies.

In addition to their frequency, the way in which reminders are provided might also play a role. In this study, the reminder was displayed as an additional screen after the ambulatory assessment. This procedure ensured that none of the experimental groups had to use the study app more frequently than the other. Still, the effects of reminders could easily be confounded with students’ use of the study app, which itself might have reminded students to study their vocabulary. Empirically we found that students in the experimental groups used the study app to roughly the same extent. In addition, we did not find that any questions on distributed practice, the strategy targeted by the study app, correlated with the frequency of using the study app. Taken together, these results suggests that the results of the group comparisons are not merely based on different extent of use of the study app.

Digital technology can be used to support self-regulated learning and allows for scalable interventions that remind learners of one-off interventions, potentially prolonging their effects^16^. At their best, these interventions target desirable learning behaviors, such as distributed practice, that can be generalized across domains^45^. Future research could employ other experimental designs conducive to the investigation of mobile interventions such as micro-randomized trials^46^. It should also focus on habit formation as it holds the promise of long-term behavior change that could make desirable learning behaviors, and subsequently positive learning outcomes, more likely. To investigate the interventions’ effect on habit formation, long-term studies should be employed^47^. In doing so, students should not simply be encouraged to study more frequently. They should also be given an explanation of why such a behavior is beneficial^48^.

To enhance the positive effects of interventions, learning design should also take into account the different needs of students^49^. Individualization, often driven by the same technological advances that lead to the application of mobile interventions^50^, should make the effects of mobile interventions and reminders even more pronounced^23,41,51^. It is important to note that differences in needs might not only result from differing cognitive and metacognitive abilities, but also the context in which learning takes place, academic motivation, and emotions^50^. For children, perceived enjoyment of mobile interventions should also be ensured as it can predict their intention to actually engage with a given intervention^52^. Ideally, these and their dynamic changes should therefore also be taken into account^53,54^. Overall, caution is needed when designing interventions to ensure that students need less and less external support, not more and more.

## Methods

### Open Practices

The intervention study reported here was preregistered on the Open Science Framework. The preregistration can be accessed at 10.17605/OSF.IO/KA2NV. The anonymized data, analysis script, and additional material is available at https://osf.io/6yn4u/?view_only=1071f46eb7f84b2b80ffc8c9cd01ba92.

### Participants

We recruited German-speaking fifth graders in the Fall of 2021. The young secondary school students were recruited through the social media platforms of 1) our institution, 2) the vocabulary learning app, and 3) a learning app that is not part of the current study. We also used email distribution lists of parents’ councils in multiple German states (Berlin, Hesse, Rhineland-Palatinate, and Saxony) and distributed flyers at a local school. In an online form, students’ legal guardians could register their children and give written informed consent to their participation. We then sent them an email with a link to an online form which provided them with a code to unlock the study app. 96 students enrolled in the current intervention study and activated both the study and the vocabulary learning app. After exclusion (compare Data Analysis), *N* = 85 students remained (*n* = 42 in the Control Group, *n* = 43 in the Reminder Group). This is slightly less than the *N* = 102 participants we aimed for based on our preregistered power analysis. The Reminder Group examined here was also included in another study. In this study, conducted by Breitwieser and colleagues^43^, it was compared to other groups that are not of interest for the present study. The students’ mean age was 10.67 years (range: 10.08 to 12.01 years, *SD* = 0.36). 50.59% of the students were female. One student went to school in Austria. All remaining students visited German schools. 71.76% of the students visited a “Gymnasium”, the highest track in German secondary education. The vocabulary learning app used in this intervention study was unknown to 85.88% of the students.

At the beginning of the intervention study, all participating students received a one-year premium account for the vocabulary learning app worth 29.99 €. This account enabled them to use all of the app’s features. In addition, they received gift vouchers for their participation. The value of those vouchers (0.50 € to 20.00 €) was based on the extent of their participation. Only the usage of the study app was rewarded. The students received diamonds whenever they answered the scheduled questions in the study app. The value of the voucher they received after the study was based on the number of diamonds they had collected. The usage of the vocabulary learning app was not incentivized. Ethics approval was obtained from the DIPF | Leibniz Institute for Research and Information in Education ethics committee (approval number DIPF_EK_2021_33).

### Design

**Fig. 3:**
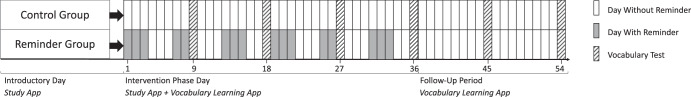
Intervention study design. A visual overview of the study design picturing the two experimental groups.

The 55 days of the intervention study were split in three phases: (1) An introductory day, (2) a 36-day intervention phase, and (3) an 18-day follow-up phase (compare Fig. 3). During the intervention phase, students used two apps, our study app and a vocabulary learning app. During the follow-up phase, only the vocabulary learning app was used. For the intervention phase, students were randomly assigned to one of two groups (Control Group or Reminder Group). Both of these groups watched a video on distributed practice on the introductory day. While the Control Group received no intervention in addition to the video, the Reminder Group received reminders of the content of the video on distributed practice on 16 of the intervention study days.

On the introductory day, students were asked to divide the vocabulary they wanted to study into six lists of about 40 word pairs from their own textbooks. These lists were uploaded into the vocabulary learning app. If students included less than 40 word pairs, vocabulary from textbooks for grade six was added. They were asked to study each list for eight days and to then take a test on the list - all in the vocabulary learning app. Thus, eight days were “study days” and a “test day” took place on the ninth day (Fig. 3). These nine days formed a study-test cycle. At the end of the cycle, the test day, students were reminded through the study app that it was time to activate a new list and the previous cycle of studying and testing was completed. For each new list, the procedure remained the same: Study the list for eight days, then take a test on the ninth day. Over the 36-day intervention phase students were supposed to follow this procedure four times, leaving us with four study-test cycles.

The 18-day follow-up period contained two study-test cycles. Once students entered the follow-up phase, they were no longer supposed to use the study app, except to complete a final questionnaire on the last day. For the usage of the vocabulary learning app, no change in instructions occurred. Students were still supposed to study their vocabulary at their own discretion and to take tests every ninth day. While this design and progression through the study’s phases was explained to the students, they differed in their adherence to it.

Students’ progression through this design generated logfile data we used to test our hypotheses. Our dependent variable for hypotheses 1 and 2 was whether students had studied vocabulary on a particular day. We considered students to have studied when they interacted with at least one vocabulary word. For hypothesis 1, which compared days with and without reminders for the Reminder Group, the independent variable was whether students saw a reminder on a particular day or not. To test hypothesis 2, we compared the two experimental groups’ study probability. Hypotheses 3 and 4 focused on the vocabulary tests students took. The dependent variable was the percentage of vocabulary words tested that was remembered correctly. To test hypothesis 3, we compared the experimental groups’ test performance. To test hypothesis 4, the independent variables used to predict test performance were the number of days students studied (i.e., interacted with at least one vocabulary word) and the amount of vocabulary they had studied.

### Apps Used in the Intervention Study

Our study app was used to explain the intervention procedure, display questions, and deliver the interventions. The app sent out notifications reminding students to use it every day. It also contained a reward system: students collected diamonds they could use to make small customizations to the study app (Supplement A).

The vocabulary learning app “cabuu” is aimed at students in grades four to seven (https://www.cabuu.app/). Within cabuu, the young students can study their own vocabulary using different study methods, such as answering multiple choice questions and performing gestures linked to specific vocabulary (see Supplement B). Cabuu allows students to create learning plans with fixed end dates. An algorithm then creates a study schedule including all vocabulary included in the learning plan. Mistakes while studying lead to a more frequent presentation of the respective vocabulary word to offer more practice (i.e., students will encounter vocabulary words more often if they did not remember them correctly before). In addition, students can test how well they learned their vocabulary using the built-in vocabulary tests. As part of the intervention study, the participating students received a code that gave them free access to the premium version of the app for one year.

### Procedure

On the introductory day, students only used the study app and prepared the vocabulary learning for its future use. They were introduced to the functions of the study app and instructed to use it every day to answer questions about their learning (ambulatory assessment). Afterwards, they were presented with questions on their study behavior, self-regulated learning, and motivation.

In addition to these instructions, students watched a video on distributed practice. The video used monsters studying for a vocabulary test as an example to illustrate what distributed practice means and what its benefits are. The video encouraged students to try and study vocabulary every day.

Each day during the intervention phase, all students received a notification at 6:30 am, reminding them to use the study app to complete the ambulatory assessment. The questions to be answered changed depending on whether a vocabulary test or regular study were planned for the day.

**Fig. 4:**
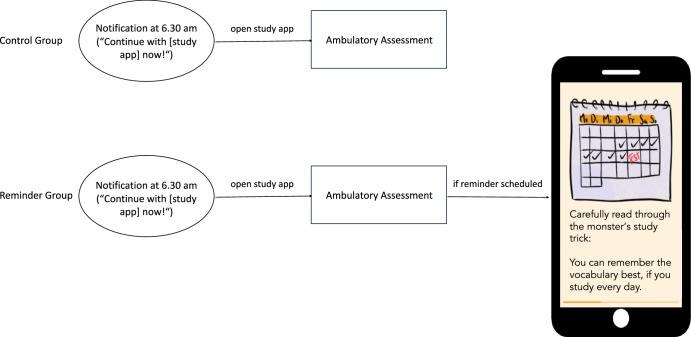
Study app routine during the intervention phase. The intended study app routine for the Control Group (upper panel) and Reminder Group (lower panel), picturing the reminder the Reminder Group was shown. The original text on the reminder screen was in German (“Lies dir den Lerntrick des Monsters genau durch: Du kannst dir die Vokabeln am besten merken, wenn du jeden Tag lernst.“).

Students in the Reminder Group received a reminder on half of the days they were supposed to study. They either started out with three consecutive days with reminders or without reminders (two reminder schedules counterbalanced across participants). The reminder schedule which switched between two and three consecutive reminders was supposed to make it harder for them to anticipate whether they would receive a reminder that day^22^. The reminder took the form of an additional screen that followed the ambulatory assessment, thus students in the Reminder Group did not need to use the app more often nor did they receive more notifications than students in the Control Group. The screen contained a screenshot of the video on distributed practice and a short text reminding students that they would get the best study outcomes if they studied every day (as shown in Fig. 4).

Thus, students could receive two sorts of reminders through the study app (see Fig. 4). All students received a notification which reminded them to use the study app (“Continue with [study app] now!“). Some students were also reminded of the contents of the video on the benefits of distributed practice. This reminder was not delivered through a push notification but instead as a screen in the study app they saw after they filled in the ambulatory assessment in the morning. This also meant that students who did not use the study app on a particular day could not receive a reminder that day – whether one was scheduled for them or not.

Over the course of the whole intervention study, participating students studied their vocabulary at their own discretion. The only instruction they received was directed at the completion of the tests scheduled every ninth day and to move on to their new learning list whenever they completed a study-test cycle.

### Data Analysis

All analyses were performed in R^55^ (Version 4.0.3). P-values reported refer to a likelihood-ratio test comparing the model including the predictor of interest to a model not including said predictor. All models used to test our hypotheses were based on logfile data from the study app and the vocabulary learning app.

As was specified in the preregistration, exclusion of participants was finalized based on the data at hand but before analyses pertaining to the hypotheses were performed. We excluded participants if they a) participated on less than five of the intervention study days of the first study-test cycle or b) if they participated on less than nine days during the 36 intervention study days. This criterion was changed from the preregistration. While we originally planned to only include students who participated on each of the first nine days, this criterion was adapted due to the fact that many students did not do so, but often still used the app quite frequently over the intervention phase. Therefore, we opted for the additional criterion of at least five days in the first study-test cycle, allowing us to still compare days with and without reminders. Based on this criterion, we excluded 10 students. In addition, we excluded one participant who only used the vocabulary learning app once. In total, our criteria led us to exclude 11 students from further analyses. Our logfile data confirmed that all students watched the video on distributed practice. We excluded data points occurring before the introductory session took place and, for students experiencing difficulty with the study app, we corrected which days we considered as their “test days”.

Some changes to the group names and order of hypotheses were made compared to the preregistration. The Control Group corresponds to Group 1 and the Reminder Group corresponds to Group 2 in the preregistration. Hypothesis 1 corresponds to hypothesis H2 and hypotheses 2 and 3 correspond to hypotheses 1a and b in the preregistration.

To test the first two hypotheses, we used logistic mixed-effects models with the binary variable “Learning Event” as the dependent variable. This variable indicates whether a student studied (1) or did not study (0) in the vocabulary learning app on a given day. We only took into account days for which no vocabulary test was scheduled. We considered students to have studied whenever they interacted with at least one vocabulary word in the vocabulary learning app. In addition, for H2 which included comparisons between the experimental groups pertaining to the reminders, we checked whether the extent of usage of the study app was roughly equivalent.

First, we tested the hypothesized within-person effect of the digital study reminders, i.e., we tested whether students in the Reminder Group were more likely to study on days they received a reminder than on days they did not (H1). Observations were included if students actually saw the reminders and did so before they studied. Days on which students were scheduled to see a reminder but did not see it in the app were considered as days without a reminder. To account for the nested data structure, we used a logistic mixed-effects model including random intercept and slope. The independent variable was the variable “Reminder” which indicated whether students received a reminder that day (1) or not (0).

Level 1:

1 $$\log\left(\frac{P({Learning}\; {Event}_{{ij}}=1)}{1-P({Learning}\; {Event}_{{ij}=1})}\right)={\beta }_{0{\rm{j}}}+{\beta }_{1j}\cdot {{\rm{Reminder}}}_{{\rm{ij}}}+{{{\varepsilon }}}_{{ij}}$$

Level 2:

2 $${\beta }_{0j}={\gamma }_{00}+{\upsilon }_{0j}$$

3 $${\beta }_{1j}={\gamma }_{10}+{\upsilon }_{1j}$$

To investigate whether students in the Reminder Group were more likely to study than those in the Control Group (H2), the binary variable “Group” (Reminder vs. Control) was included as an independent variable.

As an exploratory analysis, we compared the Reminder Group and Control Group in more detail by comparing subsets of days in the Reminder Group (with/without reminder) to a subset of days in the Control Group. This analysis was added because descriptive statistics showed that, on average, students in the Control Group were more likely to study than students in the Reminder Group, especially on days when the latter did not receive reminders. We aimed to mirror the counterbalanced orders of the reminders in the Reminder Group in the Control Group. To this end, we first used an odd-even split to split the Control Group in half. Then, for each half of the Control Group, one of the two reminder orders in the Reminder Group was used to select mock reminder days for the Control Group. This left us with similar amounts of observations for both groups for this more detailed group comparison. Here, again, the between-subjects factor was “Group” (Control vs Reminder).

Finally, we explored changes of the effects of reminders over time. For the Reminder Group, we looked into the within-person effect of the presence of a reminder over time, including an interaction term for the presence of a Reminder and the study-test cycle. For the more detailed between-group comparison, we included the group and day type (with/without reminders), using the Control Group as our reference. To test for differences in change over time, we included an interaction-term for day type (reminder vs no reminder) and day to Eq. (1).

For hypotheses 3 and 4, we took a closer look at students’ test performance. Upon first inspection of the data, we noticed that students differed in their adherence to the test schedule and the tests’ lengths. Some students skipped tests, took multiple tests within a study-test-cycle or even in the same day, making the test data very noisy. We looked into all tests students took and determined which one we would base their performance on. If students took a test earlier or later than intended, this test was counted. We included such tests if they were taken no more than three days earlier or later than planned and if they had a length of at least five tested words. If more than one test was taken within a given study-test cycle, all test activities outside of the relevant test were considered study activity. Tests taken on the same day and on the same vocabulary list were combined into one test. Test activities performed by the students and all the studying they did beforehand (but after the previous test and not on the same day) were combined into test cycles. One student was excluded from the analyses because they had studied almost 3000 vocabulary words, by far exceeding the other students’ count.

To test whether students in the Reminder Group performed better in the vocabulary tests (H3), we used a linear mixed-effects model with the percentage of vocabulary words remembered correctly as the dependent variable and “Group” (Reminder vs. Control) as a between-subjects factor. To account for the different lengths of the tests (i.e., a difference in how many words were tested), we chose to add the weighting factor “Test Length” to the preregistered model.

To test whether students performed better in vocabulary tests if they studied more frequently (H4), we used the percentage of vocabulary words remembered correctly as the dependent variable within a mixed-effects model. The independent variables “Number of Learning Events” and “Amount of Vocabulary Studied” were person and grand mean centered. These variables reflect on how many days children studied at least one vocabulary word and how many words they studied. The model, however, still yielded various convergence warnings. Many students did not adhere to the intervention study procedure. Test were taken on the wrong days or not at all, leaving us with very different actual study-test cycles for students that are hard to compare to each other. At times, what students intended to be their test was not easily recognizable from the data. This noisiness of the test data in combination with the convergence warning let us deem the results of the analysis pertaining to Hypothesis 4 to be too unreliable to be reported here. However, the analysis script and results are included in the supplementary material.

### Reporting summary

Further information on research design is available in the Nature Research Reporting Summary linked to this article.

### Supplementary information

Supplementary Materials

Reporting Summary

## Data Availability

The intervention study reported here was preregistered. The preregistration can be accessed at 10.17605/OSF.IO/KA2NV. The anonymized data and additional material are available at https://osf.io/6yn4u/?view_only=1071f46eb7f84b2b80ffc8c9cd01ba92.

## Code availability

The analysis code for the study is available at https://osf.io/6yn4u/?view_only=1071f46eb7f84b2b80ffc8c9cd01ba92.
