# Supplementary material for: Smartphone-based study reminders can be a double-edged sword
Source: NPJ Sci Learn. 2024 Jun 21;9:40. doi: 10.1038/s41539-024-00253-7 (PMC11192903; doi:10.1038/s41539-024-00253-7)
Supplement: Supplementary file 1 — Supplementary Materials [file 41539_2024_253_MOESM1_ESM.pdf]

Supplementary Materials
Smartphone Reminders to Study: A Double-Edged Sword?

Contents

A: Screenshots from the study app 1

B: Impressions of cabuu 2

C: Exemplary timeline plots of participants' activities 3

D: Extracts of R Markdown File – Test Data 4

Descriptive data on vocabulary tests 5

Analyses: H2b: Group differences in vocab test performance 6

Data preprocessing for analyses on vocabulary tests: H3 7

Analyses: H3: Prediction of test performance based on the number of study sessions beforehand 8

A: Screenshots from the study app

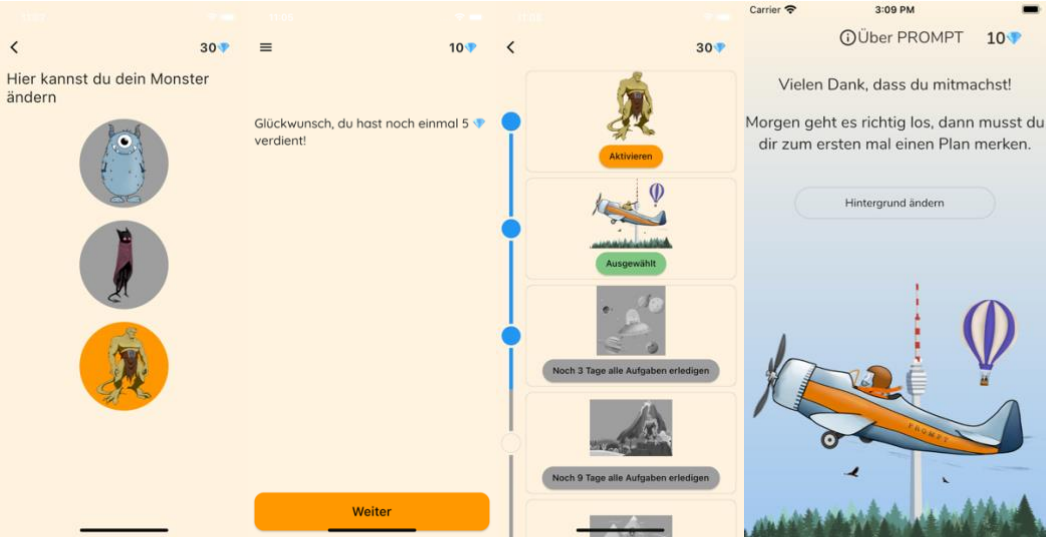

Students could choose a monster as a "study buddy" to accompany them through the app. They received diamonds for the usage of the study app. These diamonds could be used to change the app background, portraying the monster the young students chose in different situations.

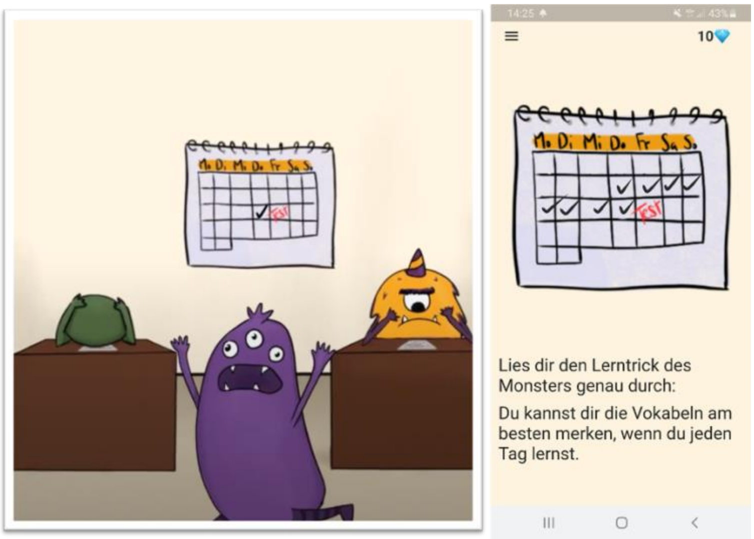

A video (screenshot on the left) explained distributed practice and its benefits based on a story on monsters preparing for a vocabulary test in school. Half of the students were regularly reminded of the strategy and its benefits through an additional screen presented in the study app (screenshot on the right).

B: Impressions of cabuu

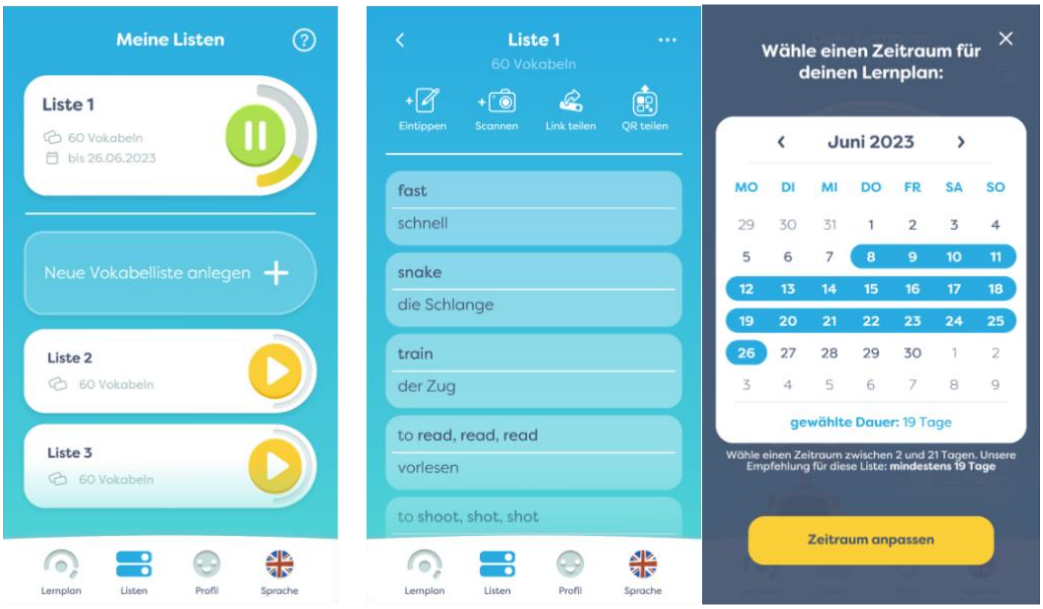

Users can create learning lists. Those lists consist of word pairs of (in our case) the German word and its English translation. Users can indicate when they want learn their list.

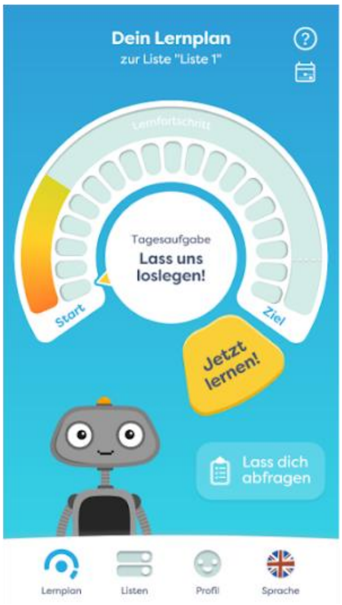

Users get learning plans and can decide whether they want to study or take a test.

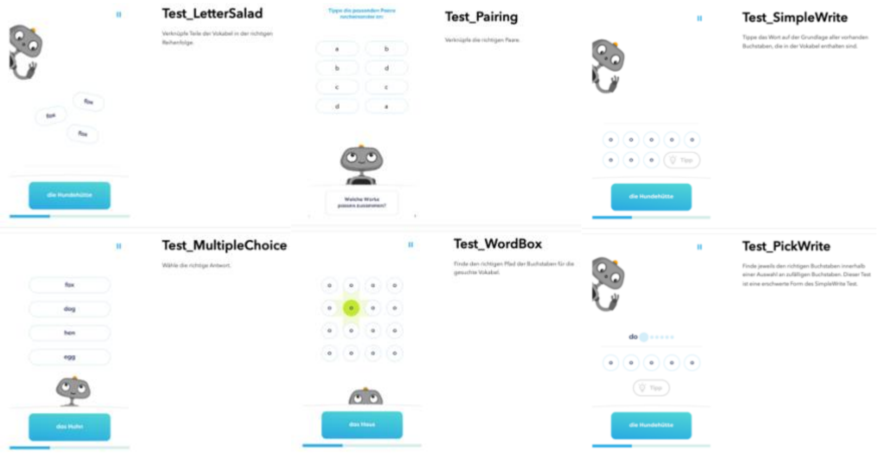

Cabuu includes different study activities.

C: Exemplary timeline plots of participants' activities

(Code for timeline plots for all participants is included in the analysis script and markdown output)

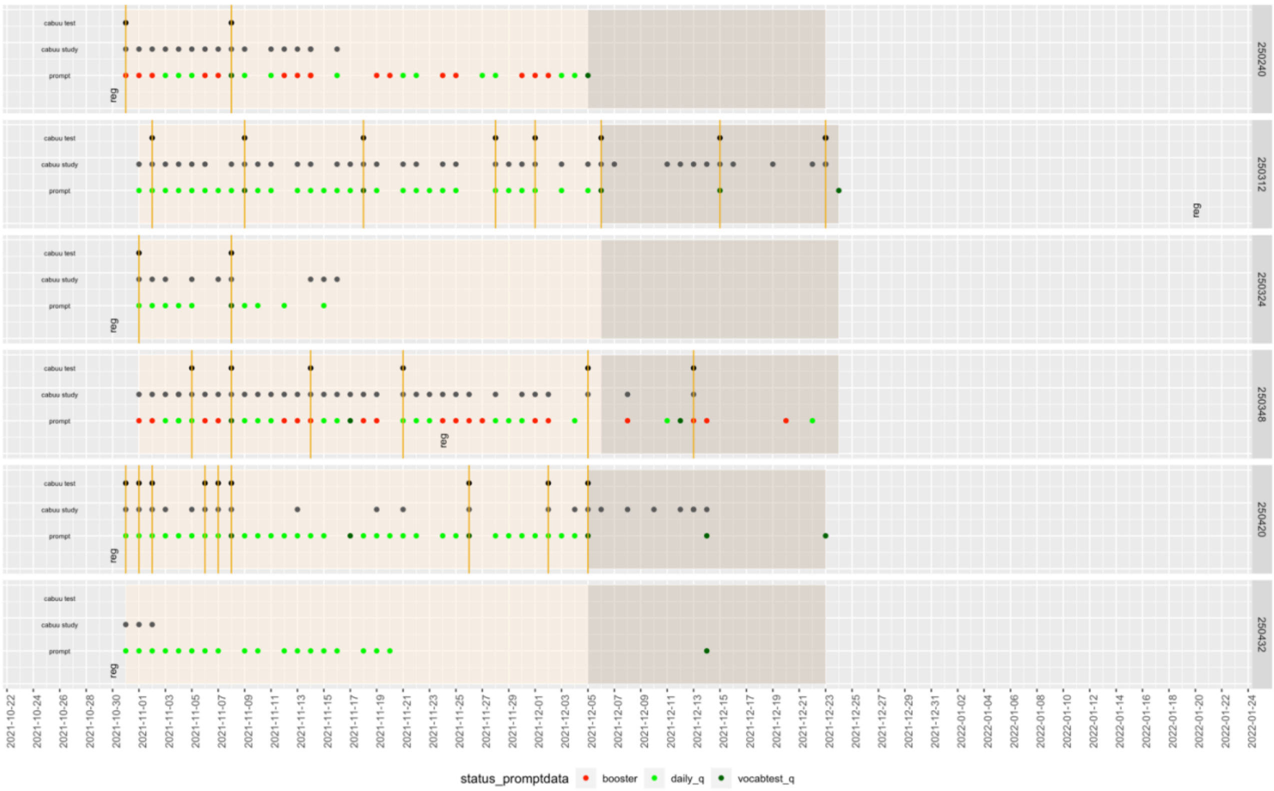

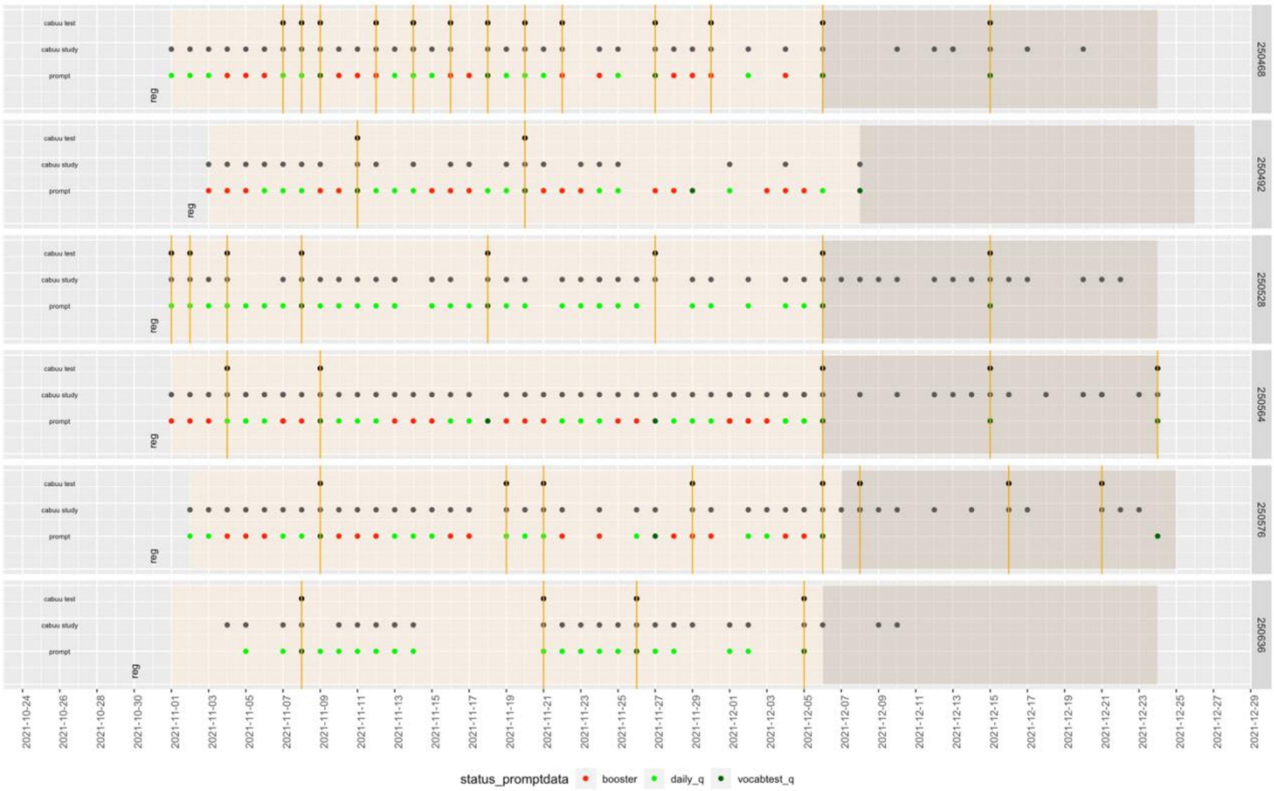

D: Extracts of R Markdown File – Test Data

The following are extracts from the full analysis script. Only test related analyses are presented here. The full script and data can be accessed at https://osf.io/6yn4u/?view_only=f2fd800493de44dc9de011bcf3dc25f9

Descriptive data on vocabulary tests

```
descriptives <- as.data.frame(aggregate(test ~ code + UserId + intervention
, first_aggregated36, sum))
#For the number of tests

summary(descriptives$test)

##    Min. 1st Qu.  Median    Mean 3rd Qu.    Max.
##   1.000   2.000   3.000   2.863   4.000   4.000

sd(descriptives$test)

## [1] 1.121941

tapply(descriptives$test, descriptives$intervention, mean)

##              dp dp + reminder
##        3.083333      2.648649

tapply(descriptives$test, descriptives$intervention, sd)

##              dp dp + reminder
##        1.130739      1.085670

descriptives <- as.data.frame(aggregate(pct_correct ~ code + UserId + inter
vention, first_aggregated36, mean))
#For test performance

summary(descriptives$pct_correct)

##    Min. 1st Qu.  Median    Mean 3rd Qu.    Max.
##  0.2581  0.6667  0.8226  0.7638  0.8991  0.9500

sd(descriptives$pct_correct)

## [1] 0.1664634

tapply(descriptives$pct_correct, descriptives$intervention, mean)

##              dp dp + reminder
##       0.7608179     0.7667033

tapply(descriptives$pct_correct, descriptives$intervention, sd)

##              dp dp + reminder
##       0.1767127     0.1582486

descriptives <- as.data.frame(aggregate(count.test ~ code + UserId + interv
ention, first_aggregated36, mean))
#For the number of vocabulary words tested

summary(descriptives$count.test)

##    Min. 1st Qu.  Median    Mean 3rd Qu.    Max.
##   10.00   29.67   38.67   37.32   43.50   84.00

sd(descriptives$count.test)

## [1] 12.25977
```

Analyses: H3: Group differences in vocab test performance

```
##Group differences in vocab test performance over intervention phase (excl
uding follow-up)
groupdiff_vocab36.null <- glmer(pct_correct  ~ 1 +  (1|code),
                               data = first_aggregated36,
                               weights = count.test, ##weigh by test size
                               family = binomial())
groupdiff_vocab36 <- glmer(pct_correct  ~ intervention +  (1|code),
                           data = first_aggregated36,
                           weights = count.test, ##weigh by test size
                           family = binomial())
summary(groupdiff_vocab36)

## Generalized linear mixed model fit by maximum likelihood
##  (Laplace Approximation) [glmerMod]
## Family: binomial  ( logit )
##Formula: pct_correct ~ intervention + (1 | code)
##   Data: first_aggregated36
##Weights: count.test

##      AIC      BIC   logLik deviance df.resid
##   1467.4   1477.4   -730.7   1461.4      206

##Scaled residuals:
##     Min      1Q  Median      3Q     Max
##-5.7463 -0.8656  0.0074  1.1148  4.0685

##Random effects:
## Groups Name        Variance Std.Dev.
## code   (Intercept) 0.7419   0.8614
##Number of obs: 209, groups:  code, 73

##Fixed effects:
##                            Estimate Std. Error z value Pr(>|z|)
##(Intercept)                   1.3954     0.1526   9.143   <2e-16 ***
##interventiondp + reminder    0.0260     0.2147   0.121    0.904
##---
##Signif. codes:  0 '***' 0.001 '**' 0.01 '*' 0.05 '.' 0.1 ' ' 1

##Correlation of Fixed Effects:
##            (Intr)
##intrvntnd+r -0.710

anova(groupdiff_vocab36.null, groupdiff_vocab36)

## Data: first_aggregated36
## Models:
## groupdiff_vocab36.null: pct_correct ~ 1 + (1 | code)
## groupdiff_vocab36: pct_correct ~ intervention + (1 | code)
##                          npar    AIC    BIC  logLik deviance  Chisq Df Pr(
>Chisq)
## groupdiff_vocab36.null     2 1465.4 1472.1 -730.72   1461.4
## groupdiff_vocab36          3 1467.4 1477.5 -730.71   1461.4 0.0147  1
0.9036
```

```
se <- sqrt(diag(vcov(groupdiff_vocab36)))
(tab <- cbind(Est = fixef(groupdiff_vocab36), LL = fixef(groupdiff_vocab36)
- 1.96 * se, UL = fixef(groupdiff_vocab36) + 1.96 *
                se))

##                                    Est         LL        UL
## (Intercept)                 1.39540397  1.0962660 1.6945419
## interventiondp + reminder 0.02600249 -0.3947166 0.4467216

##Group differences in vocab test performance over complete study period (i
ncluding follow-up)
groupdiff_vocab <- glmer(pct_correct  ~ intervention +  (1|code),
                         data = first_aggregated,
                         weights = count.test, ##weigh by test size
                         family = binomial())
summary(groupdiff_vocab)

## Generalized linear mixed model fit by maximum likelihood
##  (Laplace Approximation) [glmerMod]
## Family: binomial  ( logit )
##Formula: pct_correct ~ intervention + (1 | code)
##   Data: first_aggregated
##Weights: count.test

##     AIC      BIC   logLik deviance df.resid
##  1918.0   1928.7   -956.0   1912.0      259

##Scaled residuals:
##    Min      1Q  Median      3Q     Max
##-5.7574 -0.8514  0.0564  1.1007  4.0965

##Random effects:
## Groups Name        Variance Std.Dev.
## code   (Intercept) 0.6975   0.8351
##Number of obs: 262, groups:  code, 73

##Fixed effects:
##                            Estimate Std. Error z value Pr(>|z|)
##(Intercept)                 1.32419    0.14736   8.986   <2e-16 ***
##interventiondp + reminder  0.08871    0.20742   0.428    0.669
##---
##Signif. codes:  0 '***' 0.001 '**' 0.01 '*' 0.05 '.' 0.1 ' ' 1

##Correlation of Fixed Effects:
##            (Intr)
##intrvntnd+r -0.709
```

Data preprocessing for analyses on vocabulary tests: H4

```
## -----------------------------------------------------------------------------
------

## You have loaded plyr after dplyr - this is likely to cause problems.
## If you need functions from both plyr and dplyr, please load plyr first,
then dplyr:
## library(plyr); library(dplyr)
```

```
## ----------------------------------------------------------------------------
------

##
## Attaching package: 'plyr'

## The following objects are masked from 'package:dplyr':
##
##     arrange, count, desc, failwith, id, mutate, rename, summarise,
##     summarize

## The following object is masked from 'package:purrr':
##
##     compact

## Joining by: code, UserId, index.day, Date
```

Analyses: H4: Prediction of test performance based on the number of study sessions beforehand

```
vocabperf <- glmer(pct_correct_grouped  ~ learningevent + count +  (1|code)
,
                   data = dp_test.cycle_aggregated,
                   weights = count.test_grouped, ##weigh by test size
                   family = binomial())

## Warning in checkConv(attr(opt, "derivs"), opt$par, ctrl = control$checkC
onv, :
## Model failed to converge with max|grad| = 0.00741119 (tol = 0.002, compo
nent 1)

## Warning in checkConv(attr(opt, "derivs"), opt$par, ctrl = control$checkC
onv, : Model is nearly unidentifiable: very large eigenvalue
##  - Rescale variables?;Model is nearly unidentifiable: large eigenvalue r
atio
##  - Rescale variables?

summary(vocabperf)

## Generalized linear mixed model fit by maximum likelihood (Laplace
##   Approximation) [glmerMod]
##  Family: binomial  ( logit )
## Formula: pct_correct_grouped ~ learningevent + count + (1 | code)
##    Data: dp_test.cycle_aggregated
## Weights: count.test_grouped
##
##      AIC      BIC   logLik deviance df.resid
##   1938.3   1952.6   -965.2   1930.3      259
##
## Scaled residuals:
##     Min      1Q  Median      3Q     Max
## -5.3837 -0.8583  0.0668  1.1578  4.2623
##
## Random effects:
##  Groups Name        Variance Std.Dev.
##  code   (Intercept) 0.7427   0.8618
## Number of obs: 263, groups:  code, 74
```

```
##
## Fixed effects:
##                   Estimate Std. Error z value Pr(>|z|)
## (Intercept)     1.3662445  0.1315623  10.385  < 2e-16 ***
## learningevent -0.0643499  0.0134800  -4.774 1.81e-06 ***
## count          0.0010632  0.0001588   6.693 2.19e-11 ***
## ---
## Signif. codes:  0 '***' 0.001 '**' 0.01 '*' 0.05 '.' 0.1 ' ' 1
##
## Correlation of Fixed Effects:
##             (Intr) lrnngv
## learningvnt -0.429
## count       -0.073 -0.591
## optimizer (Nelder_Mead) convergence code: 0 (OK)
## Model failed to converge with max|grad| = 0.00741119 (tol = 0.002, compo
nent 1)
## Model is nearly unidentifiable: very large eigenvalue
##  - Rescale variables?
## Model is nearly unidentifiable: large eigenvalue ratio
##  - Rescale variables?
```

```
vocabperf36 <- glmer(pct_correct_grouped  ~ learningevent + count +  (1|cod
e),
                     data = dp_test.cycle_aggregated36,
                     weights = count.test_grouped, ##weigh by test size
                     family = binomial())
```

```
## Warning in checkConv(attr(opt, "derivs"), opt$par, ctrl = control$checkC
onv, : Model failed to converge with max|grad| = 0.00350674 (tol = 0.002, c
omponent 1)

## Warning in checkConv(attr(opt, "derivs"), opt$par, ctrl = control$checkC
onv, : Model is nearly unidentifiable: very large eigenvalue
##  - Rescale variables?;Model is nearly unidentifiable: large eigenvalue r
atio
##  - Rescale variables?
```

```
summary(vocabperf36)
```

```
## Generalized linear mixed model fit by maximum likelihood (Laplace
##   Approximation) [glmerMod]
##  Family: binomial  ( logit )
## Formula: pct_correct_grouped ~ learningevent + count + (1 | code)
##    Data: dp_test.cycle_aggregated36
## Weights: count.test_grouped
##
##      AIC      BIC   logLik deviance df.resid
##   1478.0   1491.9   -735.0   1470.0      239
##
## Scaled residuals:
##     Min      1Q  Median      3Q     Max
## -5.3663 -0.7168  0.0000  0.8797  5.3640
##
## Random effects:
##  Groups Name        Variance Std.Dev.
##  code   (Intercept) 0.8082   0.899
```

```
## Number of obs: 243, groups:  code, 74
##
## Fixed effects:
##                Estimate Std. Error z value Pr(>|z|)
## (Intercept)    1.2302479  0.1561004   7.881 3.24e-15 ***
## learningevent -0.0441021  0.0172502  -2.557   0.0106 *
## count          0.0011047  0.0001769   6.245 4.25e-10 ***
## ---
## Signif. codes:  0 '***' 0.001 '**' 0.01 '*' 0.05 '.' 0.1 ' ' 1
##
## Correlation of Fixed Effects:
##             (Intr) lrnngv
## learningvnt -0.561
## count       -0.080 -0.509
## optimizer (Nelder_Mead) convergence code: 0 (OK)
## Model failed to converge with max|grad| = 0.00350674 (tol = 0.002, compo
nent 1)
## Model is nearly unidentifiable: very large eigenvalue
##  - Rescale variables?
## Model is nearly unidentifiable: large eigenvalue ratio
##  - Rescale variables?
```

```
allFit(vocabperf36)
```

```
## bobyqa :
```

```
## Warning in checkConv(attr(opt, "derivs"), opt$par, ctrl = control$checkC
onv, : Model failed to converge with max|grad| = 0.576939 (tol = 0.002, com
ponent 1)
```

```
## Warning in checkConv(attr(opt, "derivs"), opt$par, ctrl = control$checkC
onv, : Model is nearly unidentifiable: very large eigenvalue
##  - Rescale variables?;Model is nearly unidentifiable: large eigenvalue r
atio
##  - Rescale variables?
```

```
## [OK]
## Nelder_Mead :
```

```
## Warning in checkConv(attr(opt, "derivs"), opt$par, ctrl = control$checkC
onv, : Model failed to converge with max|grad| = 0.00441522 (tol = 0.002, c
omponent 1)
```

```
## Warning in checkConv(attr(opt, "derivs"), opt$par, ctrl = control$checkC
onv, : Model is nearly unidentifiable: very large eigenvalue
##  - Rescale variables?;Model is nearly unidentifiable: large eigenvalue r
atio
##  - Rescale variables?
```

```
## [OK]
## nlminbwrap : [failed]
## optimx.L-BFGS-B :
```

```
## Warning in optimx.check(par, optcfg$ufn, optcfg$ugr, optcfg$uhess, lower
, : Parameters or bounds appear to have different scalings.
##   This can cause poor performance in optimization.
```

```
##   It is important for derivative free methods like BOBYQA, UOBYQA, NEWUO
A.

## Warning in optwrap(optimizer, devfun, start, rho$lower, control = contro
l, :
## convergence code 9999 from optimx: none

## Warning in optimx.check(par, optcfg$ufn, optcfg$ugr, optcfg$uhess, lower
, : Parameters or bounds appear to have different scalings.
##   This can cause poor performance in optimization.
##   It is important for derivative free methods like BOBYQA, UOBYQA, NEWUO
A.

## Warning in optwrap(optimizer, devfun, start, rho$lower, control = contro
l, :
## convergence code 9999 from optimx: none

## Warning in optimx.check(par, optcfg$ufn, optcfg$ugr, optcfg$uhess, lower
, : Parameters or bounds appear to have different scalings.
##   This can cause poor performance in optimization.
##   It is important for derivative free methods like BOBYQA, UOBYQA, NEWUO
A.

## Warning in optwrap(optimizer, devfun, start, rho$lower, control = contro
l, :
## convergence code 9999 from optimx: none

## [failed]
## nloptwrap.NLOPT_LN_NELDERMEAD :

## Warning in checkConv(attr(opt, "derivs"), opt$par, ctrl = control$checkC
onv, :
## Model failed to converge with max|grad| = 0.00509936 (tol = 0.002, compo
nent 1)

## Warning in checkConv(attr(opt, "derivs"), opt$par, ctrl = control$checkC
onv, : Model is nearly unidentifiable: very large eigenvalue
##  - Rescale variables?;Model is nearly unidentifiable: large eigenvalue r
atio
##  - Rescale variables?

## [OK]
## nloptwrap.NLOPT_LN_BOBYQA :

## Warning in checkConv(attr(opt, "derivs"), opt$par, ctrl = control$checkC
onv, : Model failed to converge with max|grad| = 0.176572 (tol = 0.002, com
ponent 1)

## Warning in checkConv(attr(opt, "derivs"), opt$par, ctrl = control$checkC
onv, : Model is nearly unidentifiable: very large eigenvalue
##  - Rescale variables?;Model is nearly unidentifiable: large eigenvalue r
atio
##  - Rescale variables?

## [OK]

## original model:
## pct_correct_grouped ~ learningevent + count + (1 | code)
```

```
## data:  dp_test.cycle_aggregated36
## optimizers (6): bobyqa, Nelder_Mead, nlminbwrap, optimx.L-BFGS-B,nloptwr
ap.NLOPT_LN_NELDERME...
## 2 optimizer(s) failed
## differences in negative log-likelihoods:
## max= 0.0097 ; std dev= 0.00485
```

```
#without weights
vocabperf <- lmer(pct_correct_grouped  ~ learningevent + count + (1|code),
dp_test.cycle_aggregated)
summary(vocabperf)
```

```
## Linear mixed model fit by REML ['lmerMod']
## Formula: pct_correct_grouped ~ learningevent + count + (1 | code)
##    Data: dp_test.cycle_aggregated
##
## REML criterion at convergence: -125.9
##
## Scaled residuals:
##     Min      1Q  Median      3Q     Max
## -3.7735 -0.3681  0.1233  0.5573  2.0456
##
## Random effects:
##  Groups   Name        Variance Std.Dev.
##  code     (Intercept) 0.02038  0.1427
##  Residual             0.02191  0.1480
## Number of obs: 263, groups:  code, 74
##
## Fixed effects:
##                Estimate Std. Error t value
## (Intercept)    7.461e-01  3.189e-02  23.392
## learningevent -6.306e-03  4.448e-03  -1.417
## count          1.314e-04  4.693e-05   2.800
##
## Correlation of Fixed Effects:
##             (Intr) lrnngv
## learningvnt -0.624
## count       -0.027 -0.588
```

```
vocabperf36 <- lmer(pct_correct_grouped  ~ learningevent + count +  (1|code
), dp_test.cycle_aggregated36)
summary(vocabperf)
```

```
## Linear mixed model fit by REML ['lmerMod']
## Formula: pct_correct_grouped ~ learningevent + count + (1 | code)
##    Data: dp_test.cycle_aggregated
##
## REML criterion at convergence: -125.9
##
## Scaled residuals:
##     Min      1Q  Median      3Q     Max
## -3.7735 -0.3681  0.1233  0.5573  2.0456
##
## Random effects:
##  Groups   Name        Variance Std.Dev.
##  code     (Intercept) 0.02038  0.1427
```

```
##  Residual             0.02191  0.1480
## Number of obs: 263, groups:  code, 74
##
## Fixed effects:
##                 Estimate Std. Error t value
## (Intercept)    7.461e-01  3.189e-02  23.392
## learningevent -6.306e-03  4.448e-03  -1.417
## count          1.314e-04  4.693e-05   2.800
##
## Correlation of Fixed Effects:
##              (Intr) lrnngv
## learningvnt -0.624
## count       -0.027 -0.588
```

```
library(nlme)
vocabperf2 <- lme(pct_correct_grouped  ~ learningevent.smean_GMcen + learni
ngevent_scen + count.smean_GMcen + count_scen,
                  random= ~1|code,
                  data = dp_test.cycle, method="ML")
#weights = count.test, ##weigh by test size
#family = binomial())
summary(vocabperf2)
```

```
## Linear mixed-effects model fit by maximum likelihood
##   Data: dp_test.cycle
##         AIC      BIC   logLik
##   -3038.929 -2998.42 1526.465
##
## Random effects:
##  Formula: ~1 | code
##         (Intercept)  Residual
## StdDev:   0.1697178 0.1206692
##
## Fixed effects:  pct_correct_grouped ~ learningevent.smean_GMcen + learni
ngevent_scen +      count.smean_GMcen + count_scen
##                               Value   Std.Error   DF  t-value p-value
## (Intercept)               0.7454213 0.019995544 2331 37.27937  0.0000
## learningevent.smean_GMcen -0.0344450 0.018947515 2331 -1.81792  0.0692
## learningevent_scen         0.0015901 0.008142735 2331  0.19527  0.8452
## count.smean_GMcen          0.0019801 0.000162554 2331 12.18103  0.0000
## count_scen                 0.0000016 0.000056974 2331  0.02886  0.9770
##  Correlation:
##                           (Intr) lr._GM lrnng_ cn._GM
## learningevent.smean_GMcen  0.044
## learningevent_scen        -0.028 -0.051
## count.smean_GMcen         -0.036 -0.537  0.013
## count_scen                 0.001  0.020 -0.388 -0.042
##
## Standardized Within-Group Residuals:
##         Min          Q1         Med          Q3         Max
## -4.05870462 -0.47416175  0.02386884  0.50759739  3.03308146
##
## Number of Observations: 2409
## Number of Groups: 74
```

```
ggplot(data  = dp_test.cycle_aggregated,
       aes(x = learningevent,
           y = pct_correct_grouped))+
  geom_point(size = 1.2,
             alpha = .8,
             position = "jitter")+
  theme_minimal()+
  labs(title = "Learning Event & Test Performance")
```

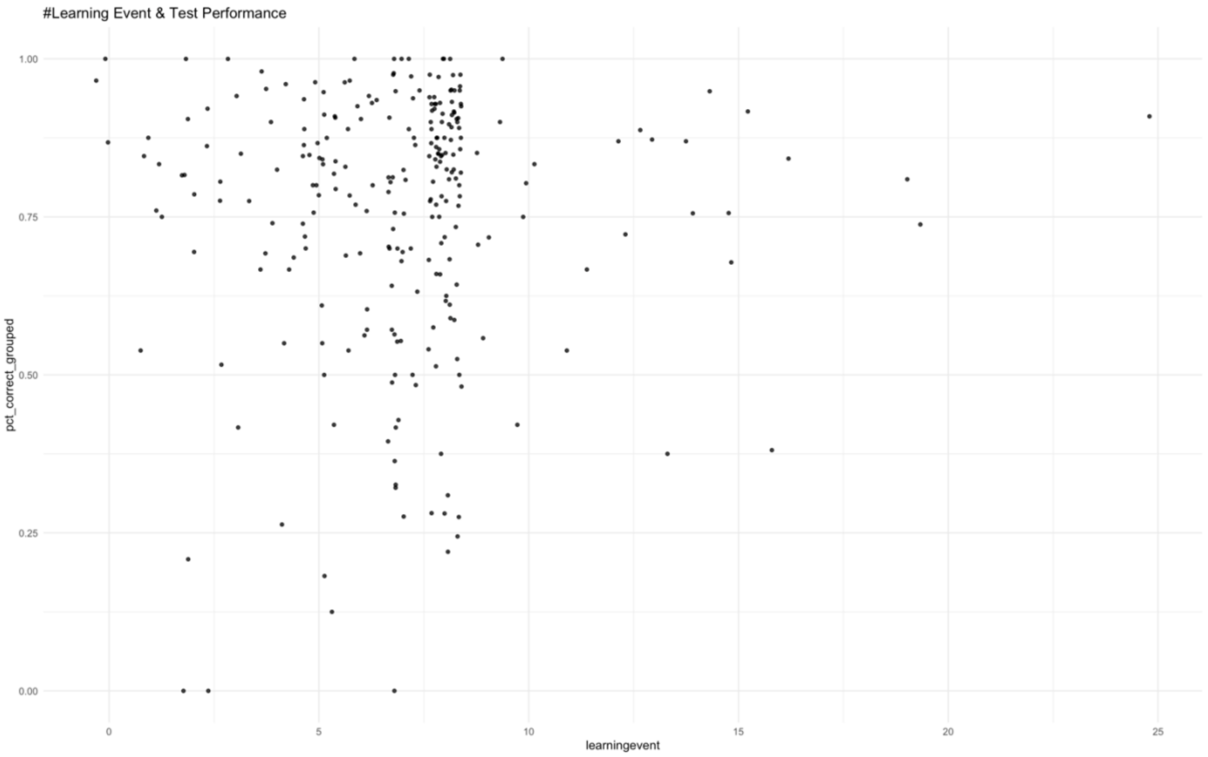

End
