# Supplementary material for: Smartphone-based study reminders can be a double-edged sword
Source: NPJ Sci Learn. 2024 Jun 21;9:40. doi: 10.1038/s41539-024-00253-7 (PMC11192903; doi:10.1038/s41539-024-00253-7)
Supplement: Supplementary file 2 — Reporting Summary [file 41539_2024_253_MOESM2_ESM.pdf]

nature portfolio

Corresponding author(s): DAPR NPJSCILEARN-01169R

Last updated by author(s): Jun 1, 2024

Reporting Summary

Nature Portfolio wishes to improve the reproducibility of the work that we publish. This form provides structure for consistency and transparency in reporting. For further information on Nature Portfolio policies, see our Editorial Policies and the Editorial Policy Checklist.

Statistics

For all statistical analyses, confirm that the following items are present in the figure legend, table legend, main text, or Methods section.

n/a	Confirmed	
☐	☒	The exact sample size (*n*) for each experimental group/condition, given as a discrete number and unit of measurement
☐	☒	A statement on whether measurements were taken from distinct samples or whether the same sample was measured repeatedly
☐	☒	The statistical test(s) used AND whether they are one- or two-sided<br>*Only common tests should be described solely by name; describe more complex techniques in the Methods section.*
☐	☒	A description of all covariates tested
☐	☒	A description of any assumptions or corrections, such as tests of normality and adjustment for multiple comparisons
☐	☒	A full description of the statistical parameters including central tendency (e.g. means) or other basic estimates (e.g. regression coefficient) AND variation (e.g. standard deviation) or associated estimates of uncertainty (e.g. confidence intervals)
☐	☒	For null hypothesis testing, the test statistic (e.g. *F*, *t*, *r*) with confidence intervals, effect sizes, degrees of freedom and *P* value noted<br>*Give P values as exact values whenever suitable.*
☒	☐	For Bayesian analysis, information on the choice of priors and Markov chain Monte Carlo settings
☐	☒	For hierarchical and complex designs, identification of the appropriate level for tests and full reporting of outcomes
☐	☒	Estimates of effect sizes (e.g. Cohen's *d*, Pearson's *r*), indicating how they were calculated

Our web collection on statistics for biologists contains articles on many of the points above.

Software and code

Policy information about availability of computer code

Data collection	We used a self-developed app to collect data and roll out our intervention. In addition, we received data from cabuu, a vocabulary learning app for children.
Data analysis	We used R (Version 4.0.3) to analyze our data.

For manuscripts utilizing custom algorithms or software that are central to the research but not yet described in published literature, software must be made available to editors and reviewers. We strongly encourage code deposition in a community repository (e.g. GitHub). See the Nature Portfolio guidelines for submitting code & software for further information.

Data

Policy information about availability of data

All manuscripts must include a data availability statement. This statement should provide the following information, where applicable:

- Accession codes, unique identifiers, or web links for publicly available datasets
- A description of any restrictions on data availability
- For clinical datasets or third party data, please ensure that the statement adheres to our policy

The intervention study reported here was preregistered. The preregistration can be accessed at https://osf.io/ka2nv/?view_only=abb38eb9e1fd4291b17af7586a0536ce. The anonymized data, analysis script, and additional material are available at https://osf.io/6yn4u/?view_only=f2fd800493de44dc9de011bcf3dc25f9.

Research involving human participants, their data, or biological material

Policy information about studies with human participants or human data. See also policy information about sex, gender (identity/presentation), and sexual orientation and race, ethnicity and racism.

Reporting on sex and gender	We only reported that "50.59% of the students were female" in our description of our sample. Neither sex nor gender were further investigated in our study.
Reporting on race, ethnicity, or other socially relevant groupings	We did not report on race or ethnicity. We did not use any variables reflecting socially relevant groupings in our analyses.
Population characteristics	We provided the mean age of our population and how many of them went to the highest track school in the German school system. ". The students' mean age was 10.67 years (Range = 10.08 to 12.01 years; SD = 0.36). 50.59% of the students were female. One student went to school in Austria. All remaining students visited German schools. 71.76% of the students visited a "Gymnasium", the highest track in German secondary education. " This information was provided in the description of the sample. None of it was used in analyses.
Recruitment	We recruited German-speaking fifth graders in the Fall of 2021. The young secondary school students were recruited through the social media platforms of 1) our institution, 2) the vocabulary learning app, and 3) a learning app that is not part of the current study. We also used email distribution lists of parents' councils in multiple German states (Berlin, Hesse, Rhineland-Palatinate, and Saxony) and distributed flyers at a local school. In an online form, students' legal guardians could register their children and consent to their participation. We then sent them an email with a link to an online form which provided them with a code to unlock the study app.<br>Parents', and therefore children's, background might have influenced who signed up for voluntary participation. We expect that a less educated sample might have studied less in general, however, the effects should not be impacted by an overall drop in the extent of usage of the vocabulary learning app.
Ethics oversight	Ethics approval was obtained from the institutional ethics committee (approval number DIPF_EK_2021_33).

Note that full information on the approval of the study protocol must also be provided in the manuscript.

Field-specific reporting

Please select the one below that is the best fit for your research. If you are not sure, read the appropriate sections before making your selection.

☐ Life sciences ☒ Behavioural & social sciences ☐ Ecological, evolutionary & environmental sciences

For a reference copy of the document with all sections, see nature.com/documents/nr-reporting-summary-flat.pdf

Behavioural & social sciences study design

All studies must disclose on these points even when the disclosure is negative.

Study description	quantitative experimental
Research sample	We recruited German-speaking fifth graders in the Fall of 2021. The young secondary school students were recruited through the social media platforms of 1) our institution, 2) the vocabulary learning app, and 3) a learning app that is not part of the current study. We also used email distribution lists of parents' councils in multiple German states (Berlin, Hesse, Rhineland-Palatinate, and Saxony) and distributed flyers at a local school. In an online form, students' legal guardians could register their children and consent to their participation. We then sent them an email with a link to an online form which provided them with a code to unlock the study app. 96 students enrolled in the current intervention study and activated both the study and the vocabulary learning app. After exclusion (compare Data Analysis), N = 85 students remained (n = 42 in the Control Group, n = 43 in the Reminder Group). This is slightly less than the N = 102 participants we aimed for based on our preregistered power analysis.The students' mean age was 10.67 years (Range = 10.08 to 12.01 years; SD = 0.36). 50.59% of the students were female. One student went to school in Austria. All remaining students visited German schools. 71.76% of the students visited a "Gymnasium", the highest track in German secondary education.<br><br>We used fifth graders as they are at a transition point where they are expected to take increasing responsibility for their own learning.
Sampling strategy	We recruited German-speaking fifth graders in the Fall of 2021. The young secondary school students were recruited through the social media platforms of 1) our institution, 2) the vocabulary learning app, and 3) a learning app that is not part of the current study. We also used email distribution lists of parents' councils in multiple German states (Berlin, Hesse, Rhineland-Palatinate, and Saxony) and distributed flyers at a local school. In an online form, students' legal guardians could register their children and consent to their participation. We then sent them an email with a link to an online form which provided them with a code to unlock the study app. 96 students enrolled in the current intervention study and activated both the study and the vocabulary learning app. After exclusion (compare Data Analysis), N = 85 students remained (n = 42 in the Control Group, n = 43 in the Reminder Group). This is slightly less than the N = 102 participants we aimed for based on our preregistered power analysis.

Data collection	We used two apps to collect data. Within our self-developed study app, we provided the intervention, explained the study procedure and had children fill in short questionnaires. In addition, we were provided with the participating children's trace data from the vocabulary app they used during the study.
Timing	The data was collected between the Fall break in 2021 and Christmas break in 2021. Only few children continued to use the study app beyond the Christmas break, due to either starting the study late or technical difficulties.
Data exclusions	As was specified in the preregistration, exclusion of participants was finalized based on the data at hand but before analyses pertaining to the hypotheses were performed. We excluded participants if they a) participated on less than five of the intervention study days of the first study-test cycle or b) if they participated on less than nine days during the 36 intervention study days. This criterion was changed from the preregistration. While we originally planned to only include students who participated on each of the first nine days, this criterion was adapted due to the fact that many students did not do so, but often still used the app quite frequently over the intervention phase. Therefore, we opted for the additional criterion of at least five days in the first study-test cycle, allowing us to still compare days with and without reminders. Based on this criterion, we excluded 10 students. In addition, we excluded one participant who only used the vocabulary learning app once. In total, our criteria led us to exclude 11 students from further analyses.
Non-participation	While students and their parents could ask for their data not to be used, there was no need for them to actively drop out of the study. Students could simply stop using the apps. However, this makes it impossible to clearly determine whether a student intended to drop out or simply forgot to use the app.
Randomization	Allocation to the experimental groups was random.

Reporting for specific materials, systems and methods

We require information from authors about some types of materials, experimental systems and methods used in many studies. Here, indicate whether each material, system or method listed is relevant to your study. If you are not sure if a list item applies to your research, read the appropriate section before selecting a response.

Materials & experimental systems

n/a	Involved in the study
☒	☐ Antibodies
☒	☐ Eukaryotic cell lines
☒	☐ Palaeontology and archaeology
☒	☐ Animals and other organisms
☒	☐ Clinical data
☒	☐ Dual use research of concern
☒	☐ Plants

Methods

n/a	Involved in the study
☒	☐ ChIP-seq
☒	☐ Flow cytometry
☒	☐ MRI-based neuroimaging

Plants

Seed stocks	*Report on the source of all seed stocks or other plant material used. If applicable, state the seed stock centre and catalogue number. If plant specimens were collected from the field, describe the collection location, date and sampling procedures.*
Novel plant genotypes	*Describe the methods by which all novel plant genotypes were produced. This includes those generated by transgenic approaches, gene editing, chemical/radiation-based mutagenesis and hybridization. For transgenic lines, describe the transformation method, the number of independent lines analyzed and the generation upon which experiments were performed. For gene-edited lines, describe the editor used, the endogenous sequence targeted for editing, the targeting guide RNA sequence (if applicable) and how the editor was applied.*
Authentication	*Describe any authentication procedures for each seed stock used or novel genotype generated. Describe any experiments used to assess the effect of a mutation and, where applicable, how potential secondary effects (e.g. second site T-DNA insertions, mosiacism, off-target gene editing) were examined.*
